# Effects of external pressure on cycling performance of silicon-based lithium-ion battery: modelling and experimental validation

**DOI:** 10.1039/d4ra05354k

**Published:** 2024-09-20

**Authors:** Kai Zhang, Yinan He, Junwu Zhou, Xinyang Wang, Yong Li, Fuqian Yang

**Affiliations:** a School of Aerospace Engineering and Applied Mechanics, Tongji University Shanghai 200092 China; b School of Intelligent Manufacturing and Control Engineering, Shanghai Polytechnic University Shanghai 201209 China yongli@sspu.edu.cn; c Materials Program, Department of Chemical and Materials Engineering, University of Kentucky Lexington Kentucky 40506 USA fuqian.yang@uky.edu

## Abstract

Controlling the stress state of electrodes during electrochemical cycling can have a positive effect on the cycling performance of lithium-ion battery. In this work, we study the cycling performance of silicon-based lithium-ion half cells under the action of pressure in a range of 0.1 to 0.4 MPa. The cycling performance of the silicon-based lithium-ion half cells increases first with increasing the pressure to 0.2 MPa and then decreases with further increasing the pressure. The analysis of the surface morphologies of cycled electrodes reveals that applying a pressure of 0.2 MPa leads to the formation of fine electrode surface with the least surface cracks after the silicon-based lithium-ion half cells are cycled for 50 times, which supports the dependence of the cycling performance of the lithium-ion half cells on the pressure. The numerical results from the single particle model reveal that applying pressure can tune the stress state in a single electrode particle and reduce the tensile stress. However, the numerical results from the two-particle model point to that applying pressure can introduce tensile stress in the electrode particles due to contact deformation. Suitable pressure applied onto a lithium-ion battery is needed in order to improve the cycling performance of the lithium-ion battery without causing detrimental effects.

## Abstract 2

Controlling the stress state of electrodes during electrochemical cycling can have a positive effect on the cycling performance of lithium-ion battery.

## Graphical abstract

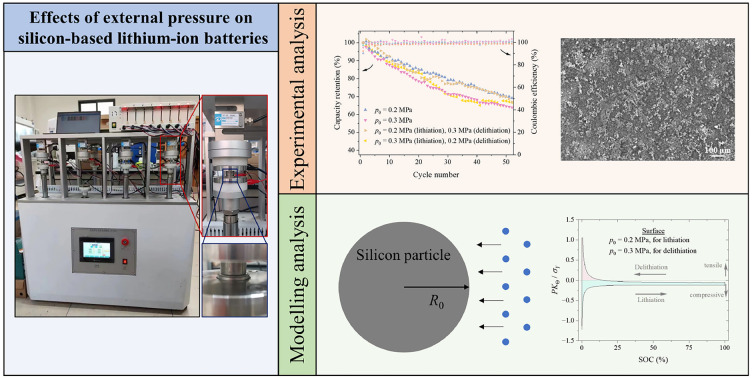

## 1 Introduction

Silicon-based energy storage systems are showing promise as potential alternatives to traditional technologies for energy storage.^1^ Compared with recently reported advanced electrode structures,^2–4^ silicon-based lithium-ion batteries (LIBs) still demonstrate superior performance with high capacity and environmental friendliness.^5–8^ The drawback with silicon-based electrodes in LIBs is the colossal volumetric change of silicon during lithiation and delithiation, which can be up to ∼300%. Such a large volumetric change can lead to mechanical and chemical damages of electrodes and capacity loss of LIBs.^9^ One of the keys to address this issue is to regulate the internal deformation and stress in silicon.^10^

One of effective ways to control the internal deformation and stress in silicon is to optimize the silicon electrode structures through structural engineering,^11^ such as producing specific nanostructures like carbon-coated core–shell,^12^ yolk–shell^13^ structures, or porous structures,^14^ fabricating advanced binders^15^ or Si/C composite electrodes,^16^ incorporating self-healing polymers and advanced prelithiation techniques. These methods improve coulombic efficiency and reduce capacity loss, thereby enhancing the durability and performance of silicon anodes.

Progress has been made in achieving favorable outcomes for silicon electrode structures, while the technical thresholds and fabrication costs have limited their commercialization. Another approach is to use specific electrochemical or mechanical loadings, *e.g.*, charging protocols and external stress to suppress the internal deformation and stress. Such an approach has also attracted much attention due to its feasibility.

Appling a partial lithiation charging protocol, Li *et al.*^17^ successfully controlled the volumetric swelling of silicon electrodes during cycling and enhanced the capacity retention of LIBs. Based on the evolution of stress in a silicon particle, Yang and co-workers proposed two stress-control charging protocols, *i.e.*, the partial delithiation method^18^ and the multi-stage currents method.^19^ Their results highlight the effectiveness of the control of internal stress in improving the cyclic performance of Si-based LIBs. Cui *et al.*^20^ investigated the effects of external pressure on the electrochemical performance of Si electrodes. They suggested that applying pressure of ∼0.6 MPa likely can reduce internal resistance and improve the capacity of the Si-based LIBs. Zhang *et al.*^21^ examined the performance of lithium-ion pouch cells with silicon-composite electrodes under pressure. The experimental results showed that the capacity of the lithium-ion pouch cells underwent slow decline followed by rapid drop under constant pressure. They observed that this issue can be mitigated by applying multi-stage pressures. Li *et al.*^22^ reviewed the effects of both external pressure and internal stress on the performance and lifespan of LIBs. They suggested that the actual effects of external pressure on the performance of LIBs depend on specific type of LIBs. In general, it is challenging to determine the optimal pressure due to the complex nature of LIBs and their responses to external mechanical loadings.

Realizing the possible effects of mechanical loading on the performance of LIBS, we investigated the cycling performance of silicon-based LIBs under the action of pressure. An *in situ* loading system was constructed and allowed for the application of pressure to the silicon-based LIBs during electrochemical cycling. The optimal pressure was determined at which the performance of silicon-based LIBs was improved. Methods of applying distinct pressures during charging and discharging were proposed to control the stress states of silicon particles during electrochemical cycling and to improve the capacity retention.

## 2 Experimental details

**Fig. 1:**
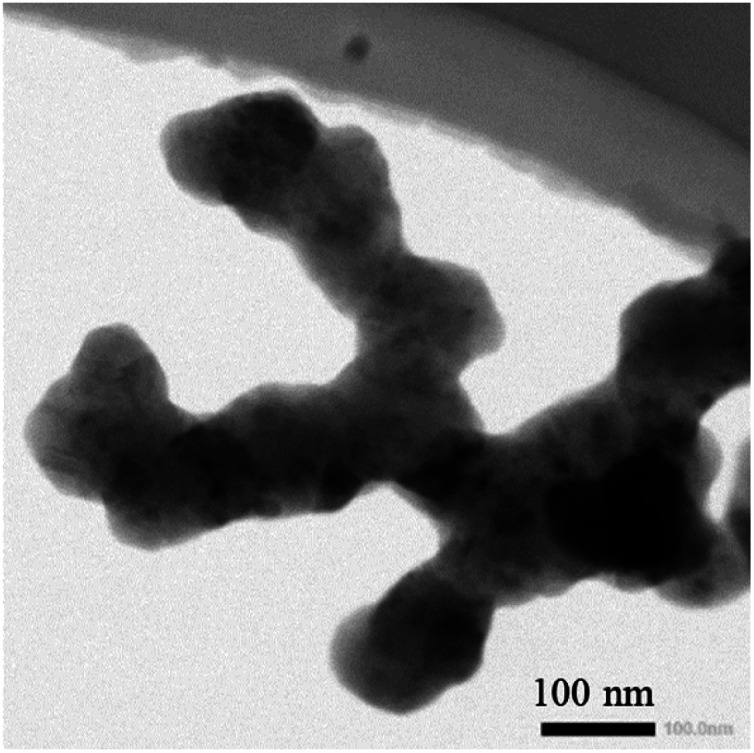
TEM image of silicon nanoparticles.

CR2032 coin half cells with Si-based working electrodes were prepared. Briefly, sodium alginate (SA, Sinopharm) as binder was dispersed in deionized water in a weight ratio of 1 : 40. Slurry consisting of 50 wt% Si nanoparticles (BTR New Energy Materials Inc.) of ∼50 nm radius (Fig. 1), 30 wt% carbon black (Super P, Timcal) and 20 wt% of the sodium-alginate suspension was prepared and stirred magnetically. The prepared slurry was spread onto a copper foil substrate (9 μm thick) to form a layer of slurry of ∼30 μm in thickness. The copper foil with the layer of slurry was dried in a vacuum oven at 110 °C for 12 h. Silicon-based electrodes of ∼12 mm were cut from the dried copper foil with the layer of slurry. Using the silicon-based electrodes and lithium foil, we assembled CR2032 coin cells in a glove box filled with an argon gas. The water vapor and oxygen in the glove box were less than 0.1 ppm. The CR2032 coin half cells used a commercial electrolyte (Tinci Materials Technology Co.) with of 1.0 M LiPF6 in EC : DEC = 3 : 7 vol% and 10 vol% FEC and a circular separators (2325, Celgard).

**Fig. 2:**
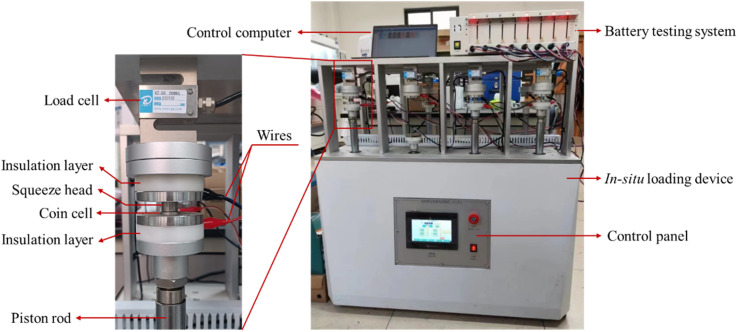
Optical image of the battery testing system with a loading system.

Electrochemical cycling of the prepared CR2032 coin half cells on a battery testing system (CT-4008Tn, Neware). The cycling tests were conducted at a C-rate of 0.2 at room temperature. The voltage window was 10–1000 mV. Prior to the tests, 5 formation cycles were performed at a C-rate of 0.1C to establish a stable SEI. The battery testing system was placed in a loading system, which was used to apply compressive force, as shown in Fig. 2. The constant load mode was used during electrochemical cycling.

After electrochemical cycling for 50 cycles, the cycled CR2032 coin half cells were disassembled in the glove box. The silicon-based electrodes were cleaned with dimethyl carbonate (DMC, Tianci Material Technology Co.) to remove contaminants and naturally dried in a glovebox for 10 h. The surface morphologies of the working electrodes were imaged on a scanning electron microscope (SEM) (S-4800, Hitachi).

## 3 Modeling

Two analytical models were developed: one is a single particle model with a single electrode particle under mechanochemical loading, and the other is a two-particle model with two electrode particles experiencing contact and fracture. In the single particle model, mechanochemical coupling was taken into account in analyzing the mechanical responses of silicon electrodes under mechanical loading and electrochemical cycling concurrently. In the two-particle model, pre-existing surface cracks were introduced. The fracture and contact behaviors of silicon particles were analyzed under pressure. The critical pressure beyond which cracks are prone to propagate was determined.

For the modeling schemes, see Appendix A.

## 4 Results and discussion

### 4.1 Experimental results

#### 4.1.1 Cycling tests

We first examine the cycling performances of the silicon-based LIBs under constant pressure. Note that a large pressure can result in short circuit inside the battery,^20^ a small pressure likely has no effect on cycling performances of the silicon-based LIBs. Four different pressures of 0.1, 0.2, 0.3 and 0.4 MPa were used in the experiments.

**Fig. 3:**
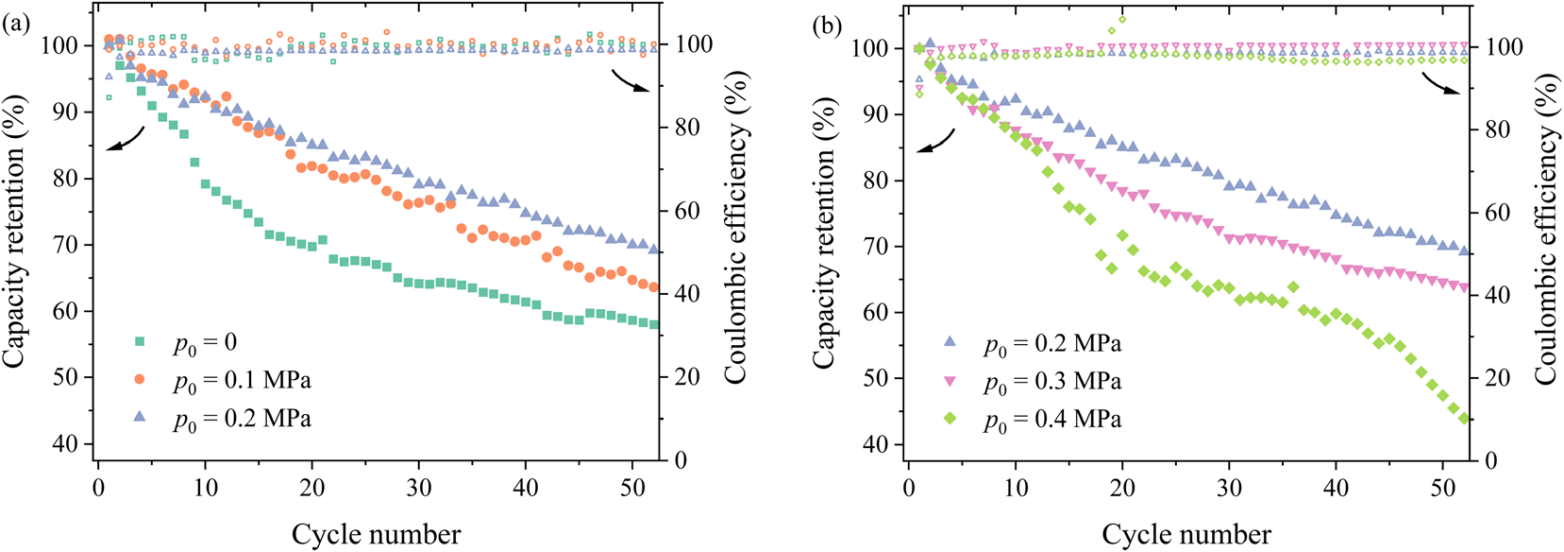
Variation of the capacity retention with the cycle number under different pressures: (a) 0–0.2 MPa and (b) 0.2–0.4 MPa.

**Fig. 4:**
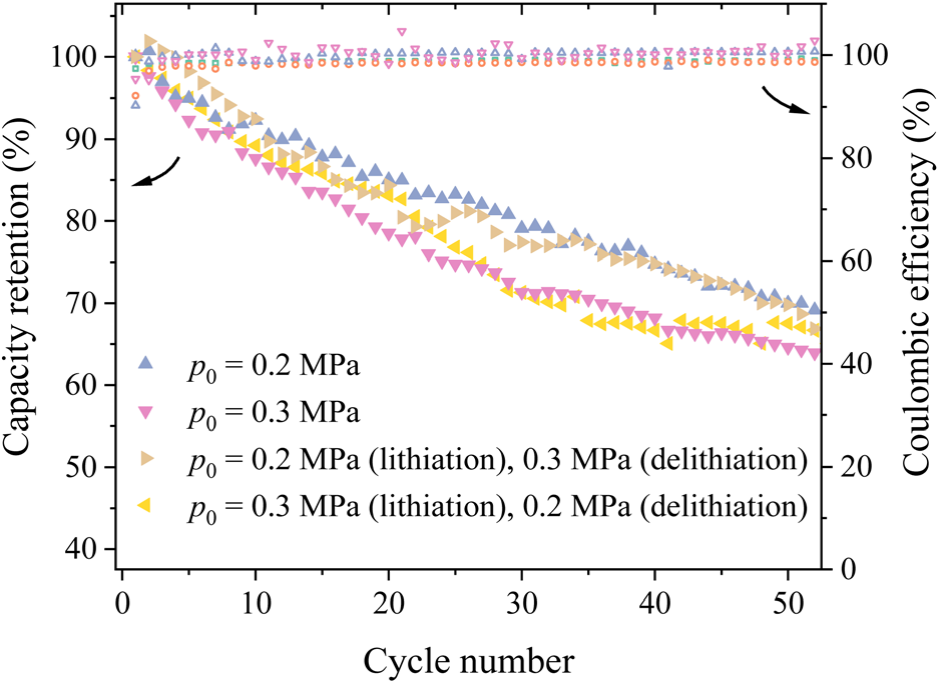
Variation of the capacity retention and coulombic efficiency with the cycle number for four different loading conditions.

Fig. 3a and b depicts the variation of the capacity retention with the cycle number under different pressures. For the same cycle number, the capacity retention increases first with increasing the of pressure, reaches maximum under the pressure of 0.2 MPa (Fig. 4a), and then decreases with increasing pressure (Fig. 4b). After the 50th cycle, the capacity retention is ∼70% under the pressure of 0.2 MPa more than ∼59% without pressure and ∼47% under the pressure of 0.4 MPa. Such a result suggests that the pressure of 0.2 MPa is likely the most suitable one applied onto the prepared silicon-based lithium-ion half cells, which does not cause detrimental effects to the lithium-ion half cells.

It is worth noting that changes in cycling performance resulting from external pressure can also be linked to the evolution of SEI layers under pressure. An increase in external pressure can promote the formation of a thin and dense SEI, enhancing the diffusivity and Young's modulus of the SEI.^23^ However, this can also lead to an increase in SEI structure delamination and a reduction in porosity.^23^ The synergistic effect of external pressure on the SEI results in a trend where the capacity retention of the battery initially increases and then decreases with the growth of external pressure, as shown in Fig. 3.

External pressure also influences the interactions among the components within the battery. Applying appropriate pressure can improve the effective contact between the battery electrode and the current collector, thereby reducing interfacial resistance and enhancing the conductivity and cycling stability. Additionally, proper pressure can strengthen the contact between the separator and electrode,^24,25^ reducing the overall internal resistance of the battery while maintaining a uniform distribution of the electrolyte within the porous electrode.^26,27^ This allows the electrolyte to fully permeate the porous structure, improving the transport efficiency of lithium ions and increasing the contact area between the active material and the electrolyte, thus enhancing the reaction efficiency of the active electrode. However, excessive pressure can damage the porous structure of the electrode material, reducing porosity, hindering lithium-ion transport, and potentially even causing the complete breakdown of the electrode structure. This explanation also aligns with the observations depicted in Fig. 3.

It has been reported that silicon electrodes can experience different stress states during lithiation and delithiation.^28^ This implies that applying different pressures during lithiation and delithiation stages likely introduces different stress states in the silicon particle during cycling. To investigate the effects of different stress states, two methods of applying pressures were used – the first one applied 0.2 MPa during lithiation and 0.3 MPa during delithiation (Method #1), and the second one applied 0.3 MPa during lithiation and 0.2 MPa during delithiation (Method #2).

Fig. 4 shows the variation of the capacity retention with the cycle number using the configurations of constant pressures of 0.2 and 0.3 MPa as well as the Method #1 and Method #2. It is evident that the silicon-based lithium-ion half cell with the configuration of Method #1 exhibited the best cycling performance for the first 10 cycles. After the first 10 cycles, the capacity retention of the lithium-ion half cells with the configuration of Method #1 decreases with increasing the cycle number and becomes comparable to the corresponding one for the lithium-ion half cells under 0.2 MPa pressure for the cycle number more than 30. The capacity retention of the lithium-ion half cells with the configuration of Method #2 experiences some fluctuations and eventually becomes slightly better than the corresponding one for the lithium-ion half cells under 0.3 MPa pressure, as evidenced by the higher capacity retention after the 50th cycle. In general, applying different pressures during lithiation and delithiation can slightly improve the cycling performance of the prepared silicon-based lithium-ion half cells. Note that there are nearly no differences in the coulombic efficiencies between the four different loading conditions.

#### 4.1.2 Surface morphologies

**Fig. 5:**
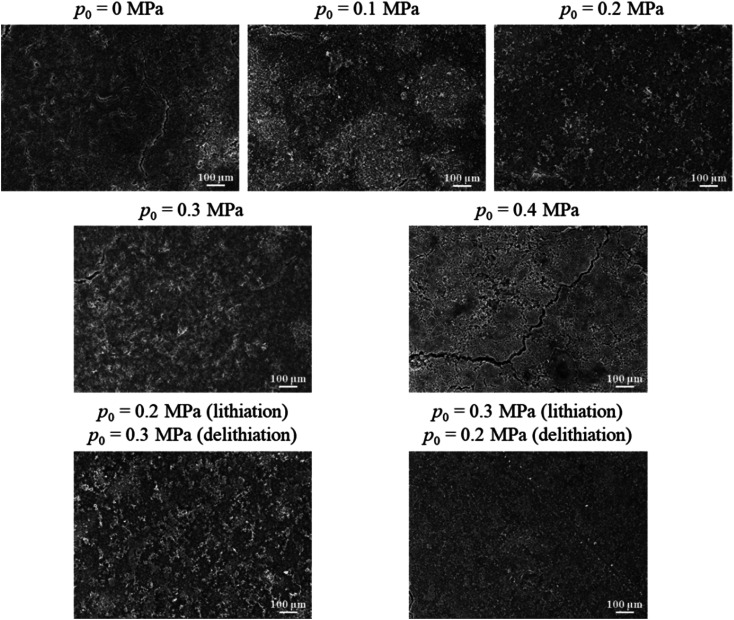
Surface morphologies of the silicon-based electrodes, which experienced 50 lithiation–delithiation cycles, under different mechanical loadings.

Fig. 5 shows SEM images of the surface morphologies of the silicon-based electrodes, which experienced 50 lithiation–delithiation cycles. There are cracks present on all the silicon-based electrodes. There are severe cracks in the surface of the silicon-based electrode under 0.4 MPa pressure and small cracks in the surface of the silicon-based electrode without pressure. Fine surface morphology is observed for the silicon-based electrode under 0.2 MPa pressure with surface cracks being visibly suppressed. The surface morphologies of the silicon-based electrodes under 0.1 and 0.3 MPa are similar, which is in accordance with the cycling performance shown in Fig. 3. The surface morphology of the silicon-based electrodes cycled with Method #1 is similar to the one cycled under 0.2 MPa pressure. The surface morphology of the silicon-based electrodes cycled with Method #2 is lightly finer than the one cycled under 0.3 MPa pressure, suggesting a slightly better cycling performance. The variation of the surface morphology of the silicon-based electrodes with the mechanical loading reveals the feasibility of controlling the internal stress state in an electrode particle by external pressure.

The diffusion-induced stress generated during cycling causes cracks to form on the surface of the silicon electrode, gradually breaking them down into powder. When the applied pressure increases to 0.1 MPa, the number of cracks on the silicon electrode significantly decreases, as the pressure enhances the contact between active material particles, promoting particle agglomeration. At a pressure of 0.2 MPa, the volume changes and pulverization of the silicon electrode are noticeably reduced, which decreases the separation of fractured silicon particles from the main body of the electrode. Additionally, the electrical contact between the active particles and adjacent units, the conductive network, and the current collector is improved, further reducing self-isolation and conductivity loss in the active material. However, when the load increases to 0.3 MPa and 0.4 MPa, particle agglomeration becomes more pronounced, but surface cracks increase, particularly for the pressure of 0.4 MPa. This is primarily because the continuously increasing external load excessively compresses the electrode structure, accelerating the fracture of silicon particles under pressure, leading to structural breakdown. As a result, more electrolyte is consumed during cycling, causing uneven SEI growth, which weakens the electrochemical reaction and accelerates capacity fading.

### 4.2 Modelling results

#### 4.2.1 Single particle model

The stress state in silicon particles plays a key role in determining the performance of silicon-based lithium-ion batteries. Applying pressure can have multifaceted effects on the performance of the silicon-based lithium-ion batteries; understanding the effects of stress state is essential to uncovering the role of pressure in controlling the cycling performance of silicon-based lithium-ion batteries.

**Fig. 6:**
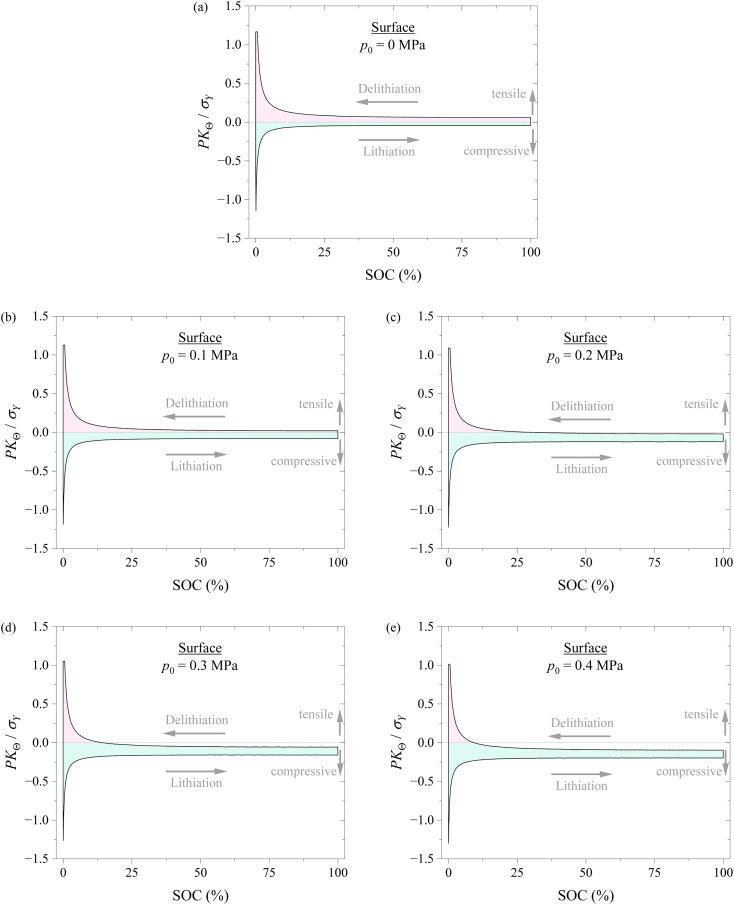
Variation of hoop stress at the surface of the spherical electrode with SOC during a lithiation–delithiation cycle under the action of different compressions of (a) 0, (b) 0.1, (c) 0.2, (d) 0.3, and (e) 0.4 MPa. The pink region corresponds to the tensile state, and the cyan region corresponds to the compressive state.

Fig. 6 shows the variation of hoop stress at the surface of the spherical electrode with SOC during a lithiation–delithiation cycle under the action of different compressions of 0, 0.1, 0.2, 0.3, and 0.4 MPa. It is evident that applying pressure causes the decrease of the hoop stress at the tensile state and the increase of the hoop stress at the compressive state for the same SOC. It is known that the hoop stress plays an important role in the nucleation and growth of surface cracks of electrodes during electrochemical cycling. The decrease in the tensile hoop stress will hinder the nucleation and growth of surface cracks and improve the structural stability of electrodes. Applying pressure might likely improve the structural stability of electrodes.

**Fig. 7:**
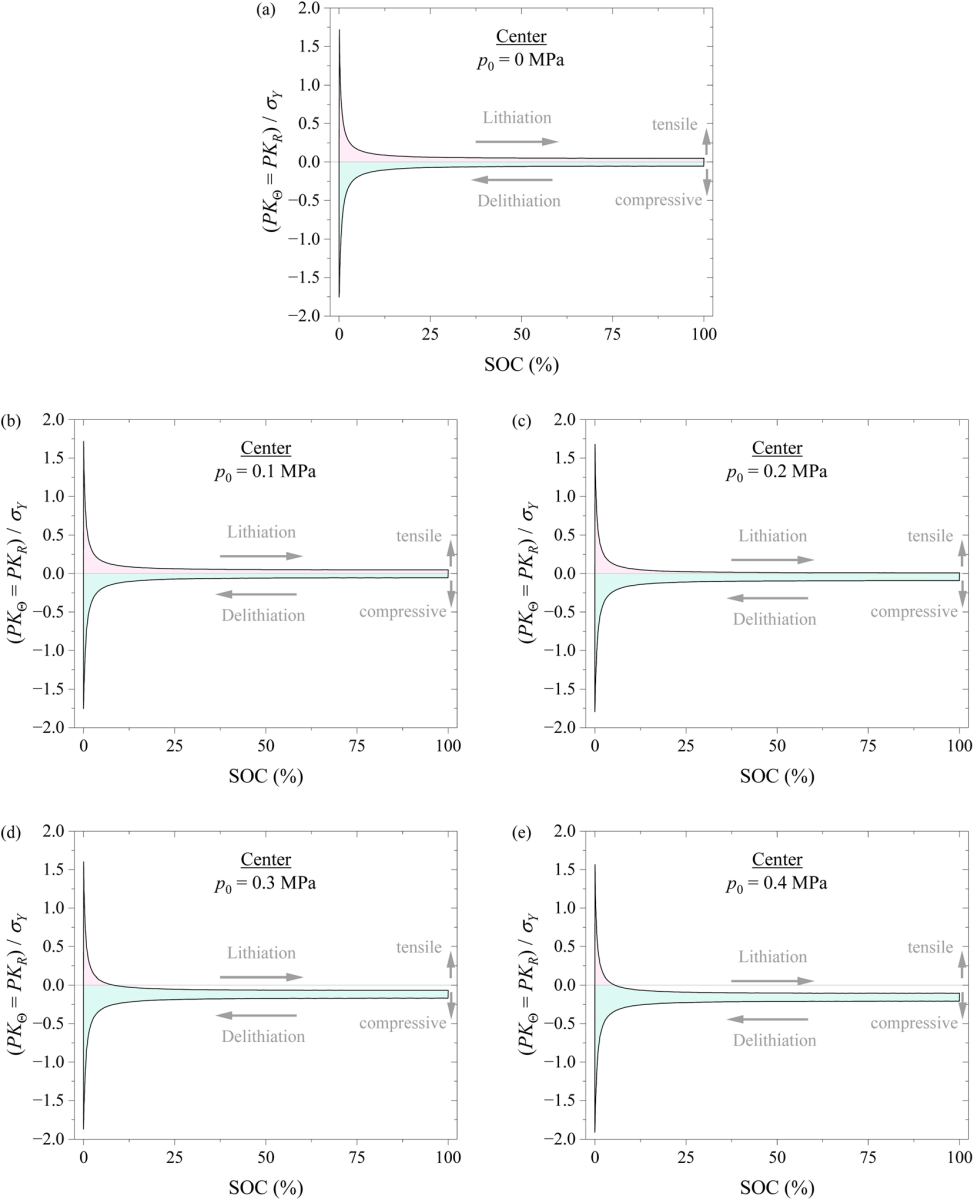
Variation of hoop stress and radial stress at the center of the spherical electrode with SOC during a lithiation–delithiation cycle under the action of different compressions of (a) 0, (b) 0.1, (c) 0.2, (d) 0.3, and (e) 0.4 MPa. The pink region corresponds to the tensile state, and the cyan region corresponds to the compressive state.

Fig. 7 shows the variations of hoop stress and radial stress at the center of the spherical electrode with SOC during a lithiation–delithiation cycle under the action of different compressions of 0, 0.1, 0.2, 0.3, and 0.4 MPa. The pink region corresponds to the tensile state, and the cyan region corresponds to the compressive state. The variations of hoop stress and radial stress at the surface of the spherical electrode with SOC exhibit similar trend to the hoop stress at the surface of the spherical electrode. Such a result also supports that applying pressure might likely improve the structural stability of electrodes.

According to the results shown in Fig. 6 and 7, it might be generally concluded that applying pressure to a battery system can likely improve the structural stability of the battery during electrochemical cycling. However, excessive pressure can lead to compressive failure of electrodes, as shown in Fig. 5. The magnitude of pressure applied to a battery system needs to be carefully controlled in order to avoid or limit the pressure-induced structural failure of electrodes and achieve a positive effect to the cycling performance of the battery system.

It should be pointed out that the stress analysis of a single electrode particle under pressure cannot provide stress state of electrodes, which consist of electrode particles, binders, and carbon blocks. A composite model needs to be developed to provide further information on the stress state of electrodes.

**Fig. 8:**
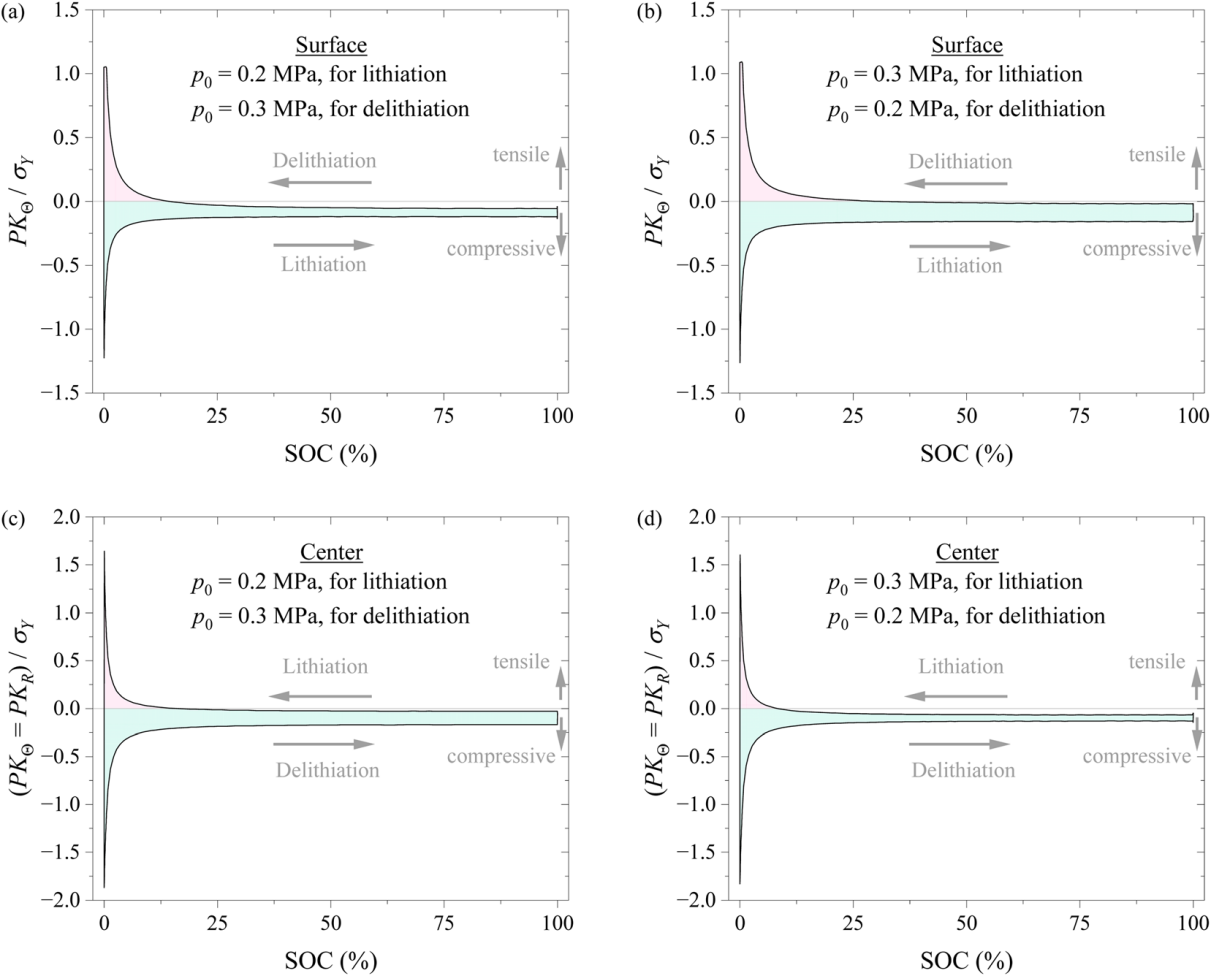
Variation of hoop stress and radial stress at the surface and at the center of the spherical electrode with SOC during a lithiation–delithiation cycle using the schemes of Method #1 and Method #2 in applying pressure. The pink region corresponds to the tensile state, and the cyan region corresponds to the compressive state. (a) Stress at the particle surface under Method #1, (b) stress at the particle surface under Method #2, (c) stress at the particle center under Method #1, and (d) stress at the particle center under Method #2.

To understand if applying different pressures during lithiation and delithiation has any impacts on the stress state of the spherical electrode, we analyzed the stress evolution in the spherical electrode with the schemes of Method #1 and Method #2 in applying pressure. Fig. 8 shows the variations of hoop stress at the surface and center of the spherical electrode with SOC during a lithiation–delithiation cycle with the schemes of Method #1 and Method #2 in applying pressure. The Method #1 reduces the stress range of the surface of the spherical electrode and magnify the stress range of the center of the spherical electrode. The opposite trend is observed for the Method #2. The experimental results given by Liu *et al.*^29^ and the analysis by Yang^30^ suggest that cracks are more prone to initiate on the surface of a silicon particle. The analysis by Zhang *et al.*^31^ reveals that the stress range is closely related to the damage of electrodes and the cycling performance of lithium-ion battery. It can be inferred that the Method #1 may be more effective in improving the cycling performance, as supported by the experimental results shown in Fig. 4. Note that the increase in the stress range at the electrode center under the scheme of the Method #1 might lead to more structural degradation at the electrode center than the scheme of the Method #2 if the nucleation of structural degradation occurs at the electrode center.

#### 4.2.2 Two-particle model

Applying pressure to electrode particles increases physical contact, reducing gaps and voids that hinder electron flow, which in turn lowers interfacial resistance and improves conductivity. This enhanced contact promotes more uniform electrochemical reactions and prevents particle isolation during cycling, maintaining efficient lithium-ion transport. Additionally, pressure helps prevent cracks and structural damage, improving the stability, longevity, and overall cycling performance of batteries. On the other hand, applying pressure can cause contact between electrode particles, which stimulates the stress state near the crack tips and triggers the propagation of surface cracks, leading to the formation of new SEI layers on the newly formed crack surfaces. This can result in the loss of active materials and the fading of capacity.

**Fig. 9:**
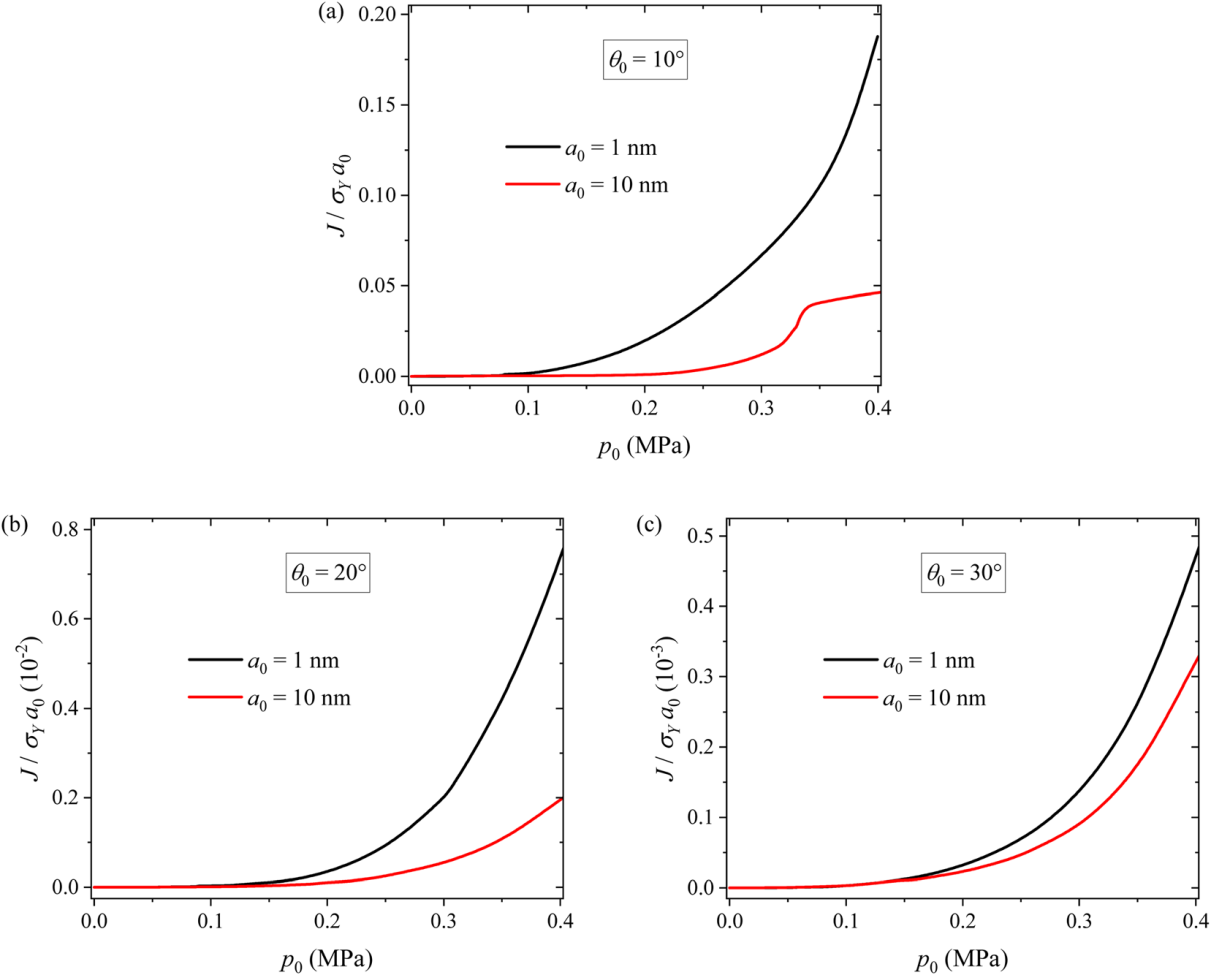
Variations of *J*-integral at the crack tip with external pressure for two different crack sizes and three different inclination angles of (a) *θ*_0_ = 10°, (b) *θ*_0_ = 20° and (c) *θ*_0_ = 30°.

To examine the potential crack propagation of electrode particles under different pressure, we calculate the evolution of the *J*-integral at the crack tip. Fig. 9 shows the variation of *J*-integral at the crack tip with applying pressure for two different crack sizes and three different inclination angles. For the same inclination angle, the *J*-integral increases with increasing pressure, as expected. Applying appropriate pressure promotes close contact between particles, reducing contact resistance and thereby improving the cycling performance of LIBs. However, it does not lead to a significant increase in the *J*-integral. This explains why the cycling test results and surface morphology of the 0.2 MPa case outperform the others. Applying large pressure can cause the propagation of surface cracks. In particular, for pressures exceeding 0.2 MPa, there is a rapid increase in the *J*-integral, resulting in the degradation of silicon particles and ultimately leading to the capacity fade of the batteries. Under the action of the same pressure, the smaller the crack size, the larger the *J*-integral. This reveals the size dependence of the surface cracking. For the same crack size, the smaller the inclination angle, the larger the *J*-integral. This result is in accord with the contact-induced ring crack in a spherical particle.^32,33^

From Fig. 9a, we note that there is a decrease in the increase rate of the *J*-integral with respect to applied pressure at *p*_0_ = ∼0.33 MPa for the crack with *a*_0_ = 10 nm and *θ*_0_ = 10°. This can be attributed to the fact that surface cracks under *θ*_0_ = 10° are located near the contact region. When the cracks are large, the contact status of the particles is greatly affected by the cracks, thus exerting a significant influence on the stress field at the crack tip and the *J*-integral. In more detail, the decrease in the increase rate of the *J*-integral is likely due to the dominance of viscoplastic flow, which consumes more mechanical work done by the applied stress. It should be pointed out that the close contact between electrode particles under pressure can reduce the contact resistance and thereby improve the cycling performance of LIBs.

## 5 Conclusions

Controlling the stress state of electrodes in a lithium-ion battery can likely improve the cycling performance of the lithium-ion battery. We demonstrated the feasibility of applying pressure onto the coin-cell shell to tune the cycling performance of the silicon-based lithium-ion half cells. The cycling performance of the silicon-based lithium-ion half cells increases first with the increase of the applied pressure and then decreases with further increasing the applied pressure. The examination of the surface morphologies of the cycled electrodes reveals the dependence of surface cracking on the applied pressure. There exists a “optimal” pressure under which the silicon-based lithium-ion half cells exhibit the “best” cycling performance and structural integrity. This result suggests that suitable pressure applied onto a lithium-ion battery can improve the cycling performance of the lithium-ion battery without causing detrimental effects.

The numerical results from the single particle model reveal that applying pressure can tune the stress state in a single electrode particle and reduce the tensile stress in the surface of the electrode particle. This can hinder the nucleation of surface cracking. However, the numerical results from the two-particle model point to that applying pressure can introduce tensile stress in the electrode particles due to contact deformation and the *J*-integral at the crack tip increases with increasing pressure. Applying large pressure can result in the propagation of surface cracks and lead to the capacity fading of lithium-ion battery. Thus, it requires suitable pressure to be applied onto a lithium-ion battery in order to improve the cycling performance of the lithium-ion battery without causing detrimental effects.

## Data availability

The data that support the findings of this study are available from the corresponding authors on reasonable request.

## Conflicts of interest

There are no conflicts to declare.

## A Appendices

### A.1 Appendix A

#### A.1.1 Modelling schemes

##### A.1.1.1 Single particle model

Lithiation of crystalline silicon leads to the phase change from crystalline phase to amorphous phase. The modeling analysis was focused on the cycling-induced deformation of amorphous silicon. A power law-based viscoplastic constitutive relationship was used in the analysis.

For the single particle model, we consider a spherical silicon particle with an initial radius of *R*_0_ in a reference coordinate system (*R*, *Θ*, *Φ*). The corresponding current coordinate system is (*r*, *θ*, *φ*). The cycling-induced deformation of the silicon particle is described by the deformation gradient tensor, ***F***, as

A.1 ***F*** = ***F***^el^***F***^pl^***F***^ch^,

with respective elastic, plastic and chemical counterparts as ***F***^el^, ***F***^pl^ and ***F***^ch^. Let ***u*** be the displacement vector. The spherical-symmetrical characteristics of the problem yields non-zero displacement of (*u*_*R*_ = *r* − *R*) in radial direction. The deformation gradient tensor of ***F*** is calculated as

A.2 ***F*** = ***I*** + diag(∂*u*_*R*_/∂*R*, *u*_*R*_/*R*, *u*_*R*_/*R*),

with ***I*** being the second rank unit tensor. The incompressible condition for plastic deformation yields det(***F***^pl^) = 1. Using the spherical-symmetrical characteristics, the plastic deformation gradient tensor of ***F***^pl^ is expressed

A.3 ***F***^pl^ = diag(*λ*_pl_, *λ*_pl_^−1/2^, *λ*_pl_^−1/2^),

where *λ*_pl_ is the radial plastic stretch. The lithiation–delithiation cycling leads to isotropic deformation of the spherical particle as

A.4 ***F***^ch^ = *Λ*^1/3^***I***,

where (*Λ* = 1 + *Ω*_1_*C*) is the volumetric expansion of the sphere induced by the intercalation of lithium into silicon, *Ω*_1_ is the volumetric strain per mole fraction of lithium, and *C* is the lithium concentration in the initial configuration. The elastic deformation gradient tensor of ***F***^el^ is obtained from eqn (A.1)–(A.4) as

A.5 ***F***^el^ = *Λ*^−1/3^diag〈(1 + ∂*u*_*R*_/∂*R*)*λ*_pl_^−1^, (1 + *μ*_*R*_/*R*)*λ*_pl_^−1/2^, (1 + *μ*_*R*_/*R*)*λ*_pl_^−1/2^〉.

The elastic strain tensor, *i.e.*, the Green-Lagrange strain tensor, ***E***^el^, can be calculated as

A.6 ***E***^el^ = diag(*E*^el^_*R*_, *E*^el^_*Θ*_, *E*^el^_*Φ*_) = [(***F***^el^)^*T*^***F***^el^ − ***I***]/2,

where *E*^el^_*R*_, *E*^el^_*Θ*_ and *E*^el^_*Φ*_ are three elastic strain components, and *E*^el^_*Θ*_ = *E*^el^_*Φ*_. Assuming that the strain energy density, *W*, is described by the Saint Venant–Kirchhoff model, we haveA.7
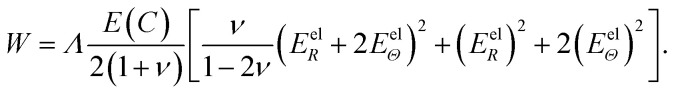
In eqn (A.7), *E*(*C*) is the concentration-dependent elastic modulus, and *ν* is Poisson's ratio. The elastic modulus is linearly dependent on the lithium concentration as

A.8 *E*(*C*) = *E*_0_(1 − *κC*/*C*_max_),

where *E*_0_ is the elastic modulus of pristine silicon, *κ* is the lithiation-induced softening coefficient, and *C*_max_ is the stoichiometric maximum concentration of lithium in silicon.

The first Piola–Kirchhoff stress tensor, ***P*** = diag(PK_*R*_, PK_*Θ*_, PK_*Φ*_), is calculated from ***P*** = ∂*W*/∂***F*** asA.9
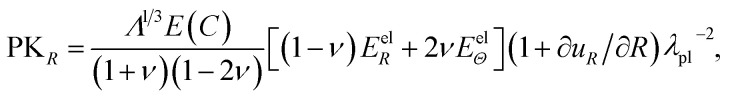
A.10
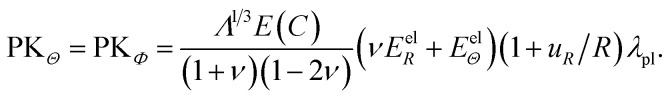


The true stress tensor, ***σ*** = diag(*σ*_*r*_, *σ*_*θ*_, *σ*_*φ*_), in the current configuration is obtained from the relation of ***σ*** = det(***F***)^−1^***PF***^*T*^. Without any body force, the mechanical equilibrium of the spherical particle is described asA.11
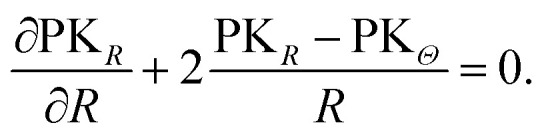


For the viscoplastic problem, we have the rate of the plastic deformation, ***D***^pl^, as

A.12 ***D***^pl^ = ***Ḟ***^pl^(***F***^pl^)^−1^ = *Λ̇*_pl_/*λ*_pl_diag(1, −1/2, −1/2).

The rate of the plastic deformation can be calculated by a plastic flow potential, *G*_pl_, as

A.13 ***D***^pl^ = ∂*G*_pl_/∂***τ***,

where (***τ*** = ***σ*** − tr(***σ***)***I***/3) is the deviatoric stress tensor. A power law-based potential for plastic flow is used in the analysis as^34,35^A.14
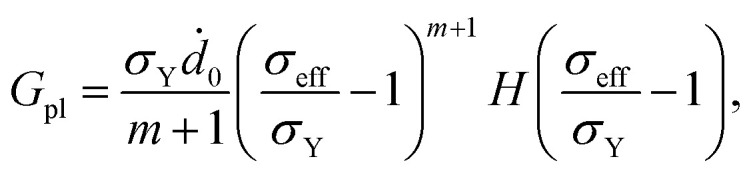
where 
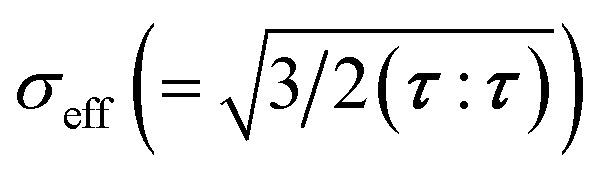
 is the effective stress, *σ*_Y_ is the yield strength, *ḋ*_0_ is the characteristic strain rate, *m* is the stress exponent for plastic flow, and *H*(·) is Heaviside function. Substituting eqn (A.12) and (A.14) into eqn (A.13), we obtain the rate equation for the cycling-induced plastic flow as

A.15 *Λ̇*_pl_/*λ*_pl_ = sgn(*σ*_*r*_ − *σ*_*θ*_)*ḋ*_0_(*σ*_eff_/*σ*_Y_ − 1)^*m*^*H*(*σ*_eff_/*σ*_Y_ − 1).

The deformation of the pristine silicon particle has initial conditions as

A.16 *u*_*R*_(*R*,0) = 0, *λ*_pl_(*R*,0) = 1.

In contrast to the works reported in literature, we take into account the effects of external mechanical load on the cycling-induced deformation of the spherical particle. Note that the external pressure cannot be applied directly to electrode, since the electrode is enclosed by coin cell case, also, the electrode itself is a porous medium filled with electrolyte. For simplification, we consider that the spherical silicon experiences a constant hydrostatic pressure exerted by an externally applied mechanical pressure, *p*_0_, through the electrolyte. This transmission pathway of forces is validated by a finite element analysis of a whole coin cell model, as demonstrated in Appendix B. Thus, the boundary conditions are

A.17 *u*_*R*_(0,*t*) = 0, PK_*R*_(*R*_0_,*t*) = *p*_0_.

With the spherical-symmetrical characteristics of the problem, there is only non-zero radial flux for mass transport, which is calculated by the spatial gradient of the chemical potential, *μ*, asA.18
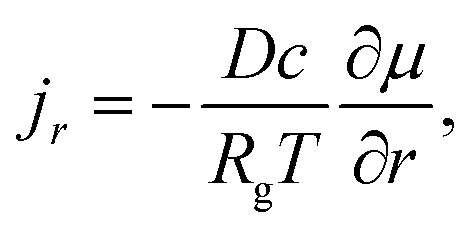
where *D* is the diffusivity, (*c* = *C*/det(***F***)) is the lithium concentration in the current configuration, *R*_g_ is gas constant, and *T* is the absolute temperature. Incorporating the contributions of the strain energy and hydrostatic stress, we have the chemical potential as^36,37^

A.19 *μ* = *μ*_0_ + *R*_g_*T* ln *c* − *Ω*_1_*σ*_*m*_ + *Ω*_2_*w*,

where *μ*_0_ is a reference value, (*σ*_*m*_ = tr(***σ***)***I***/3) is the true hydrostatic stress, *Ω*_2_ is the partial molar volume of silicon, and (*w* = *W*/det(***F***)) is the strain energy density in the current configuration. Substituting eqn (A.19) into (A.18) and expressing the diffusive flux in the initial configuration,^18,19^ one hasA.20
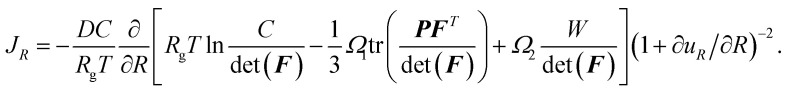


The mass conservation in the spherical particle in the reference configuration yieldsA.21
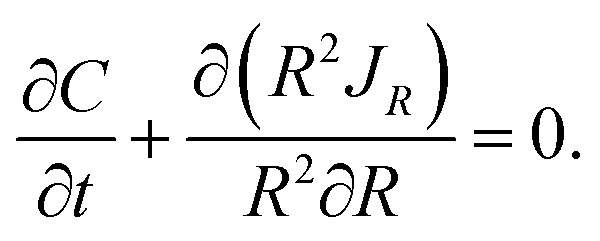


The initial condition for the pristine unlithiated silicon is

A.22 *C*(*R*,0) = 0.

To mimic the experimental conditions, we adopt galvanostatic operation in the modeling analysis and have the influx to the spherical particle asA.23
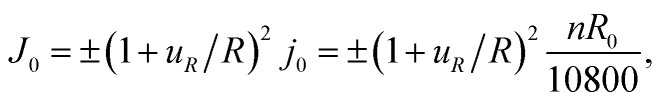
with positive values for lithiation and negative values for delithiation, and *n* the value of C-rate. To manifest the degree of lithiation, the state of charge (SOC) is calculated asA.24
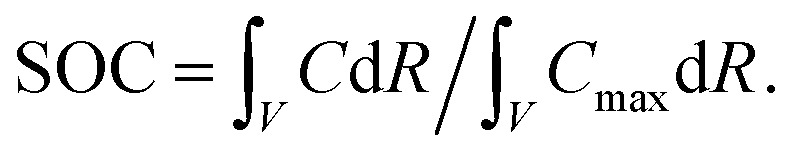


##### A.1.1.2 Two-particle model

**Fig. 10:**
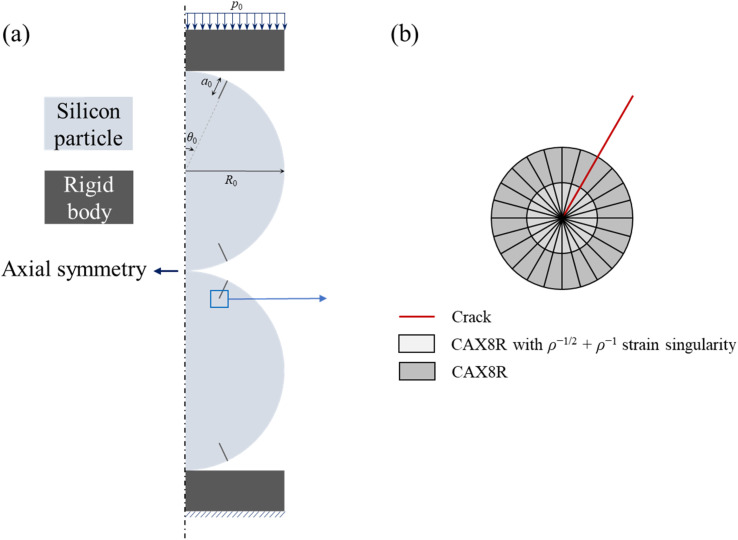
Two-particle model: (a) schematic of the axisymmetric model with geometric parameters, and (b) the mesh refinement near the crack tip.

Applying external stress can result in the compression of electrode, which may lead to contact between silicon particles. To evaluate the effects of the contact between silicon particles, a two-particle contact model, as shown schematically in Fig. 10a, was used in the modeling analysis. Pre-existing cracks on the particle surface were introduced. Two geometric parameters were considered: the angle between the crack and the axisymmetric axis, *θ*_0_, and the crack length, *a*_0_.

The compression of the two particles can cause the propagation of the pre-existing cracks, resulting in adverse effects on lithium-ion batteries, including the formation of new SEI and the capacity fading. To track the potential crack growth, we evaluate the variation of the *J*-integral around the crack tip with increasing external force. Note that the *J*-integral is not valid for the deformation with eigen strains, such as diffusion-induced strain. Here, we examine the fracture behavior of the spherical particles at the un-lithiated state. The rate-dependent viscoplastic behavior with power-law hardening was used, which is the same as the viscoplastic flow used in the single particle model of eqn (A.15). Note that for the material with a power-law flow law of eqn (A.15), one needs to consider the singularity of *ρ*^−*m*(*m*−1+1)^ at the crack tip with *ρ* as the distance from the crack tip. The *ρ*^−*m*(*m*−1+1)^ singularity cannot be created using commercial finite element method code, but the combination of *ρ*^−1^ and *ρ*^−1/2^ singularities can provide a reasonable approximation for *ρ*^−*m*(*m*−1+1)^.^38^ Therefore, the finite elements with *ρ*^−1/2^ + *ρ*^−1^ singularity were constructed at the crack tip, as shown in Fig. 10b. Quadratic axisymmetric elements with reduced integration were used in the finite element analysis. The singularity at the crack tip was achieved by collapsing the elements.

#### A.1.2 Modelling implementations

The mechanochemical model for a single particle was established and solved using the PDE module of the commercial finite element code COMSOL Multiphysics.^39^ One-dimensional spherical symmetric model was discretized into 1000 fourth-order elements. The convergence tolerance was set to 1 × 10^−9^. These ensure the accuracy of calculation.

The two-particle contact model was constructed using the commercial finite element code ABAQUS. Quadratic axisymmetric elements with reduced integration (CAX8R) were used. The contact interfaces were assumed to be frictionless. For each crack tip, 3 contour integrals of *J*-integral were collected to verify the path independence.

The material properties of silicon for both the single particle model and the two-particle model are assumed to be the same. The material properties and parameters used in the analysis are listed in Table 1.

**Table 1:** Material properties of Si and parameters used in modelling

Property/Parameter	Symbol	Value
Elastic modulus of pristine Si	*E*_0_	80 GPa (ref. 40)
Poisson's ratio	*ν*	0.29 (ref. 41)
Volumetric strain per unit mole fraction of Li	*Ω*_1_	8.18 × 10^−6^ m^3^ mol^−1^ (ref. 42)
Partial molar volume	*Ω*_2_	8.18 × 10^−6^ m^3^ mol^−1^ (ref. 42)
Yield strength	*σ*_Y_	2.6 × 10^6^ Pa (ref. 43)
Characteristic strain rate	*ḋ*_0_	0.001 (ref. 35)
Stress exponent	*m*	4 (ref. 35)
Lithiation-induced softening coefficient	*κ*	0.375 (ref. 40)
Diffusivity	*D*	1 × 10^−16^ m^2^ s^−1^ (ref. 37)
Stoichiometric maximum concentration	*C*_max_	3.67 × 10^5^ mol m^−3^ (ref. 37)
Gas constant	*R*_g_	8.3145 J K^−1^ mol^−1^
Absolute temperature	*T*	296 K
Initial radius	*R*_0_	50 nm

### A.2 Appendix B

#### A.2.1 Effect of external pressure on the hydrostatic stress in electrode structure

**Fig. 11:**
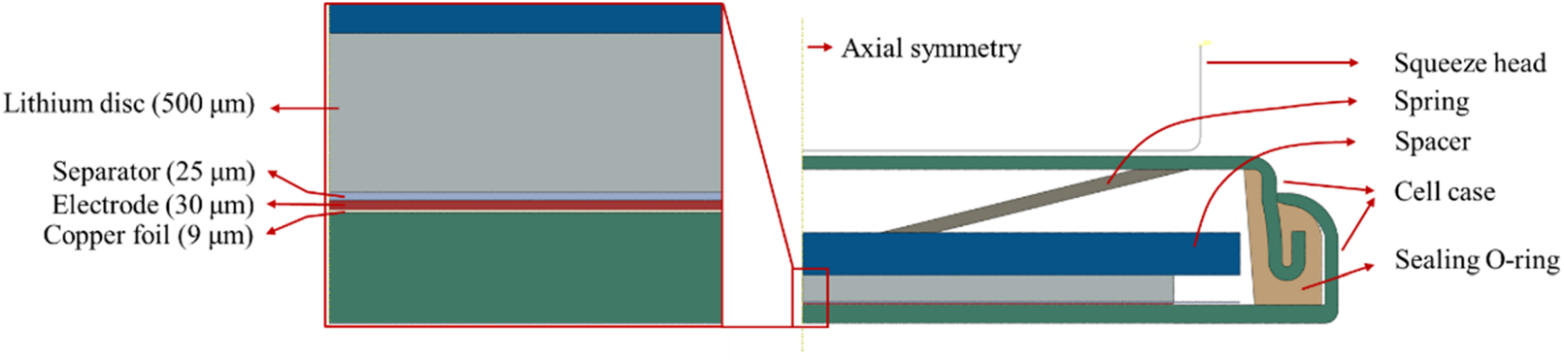
Finite element model of the coin cell under compression.

**Fig. 12:**
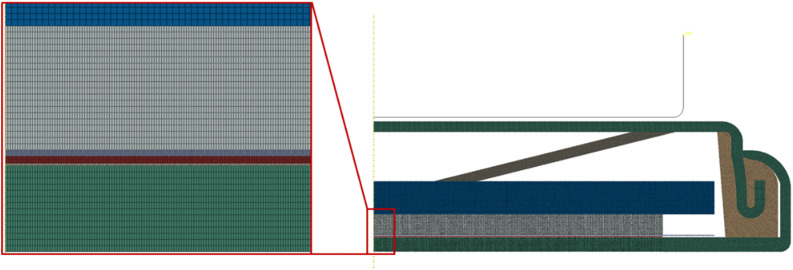
Finite mesh used in the finite element analysis.

In this work, we assume that applied mechanical pressure is transmitted to electrode through electrolyte due to the coin-cell case. To support this assumption, we performed finite element analysis. The finite element model consists of all the components of a coin cell, as shown in Fig. 11. With axisymmetric characteristics of the coin cell, an axial symmetric model was constructed. The squeeze head of 10 mm in radius was treated as a rigid body, and the deformation of the components in the coin cell is assumed to be linearly elastic. Four-node axisymmetric elements were used for the finite element model. Fine meshes were used in the regions of the separator, electrode and copper foil, as depicted in Fig. 12. Table 2 lists the material properties used in the calculation. Contacts were established between different components, and perfect bonding between the electrode and the copper foil was used. During simulation, the squeeze head moves downwards to apply mechanical pressure. Mechanical load was then transmitted through the spring, spacer, lithium foil, separator and towards the electrode.

**Table 2:** Material properties of the components of the coin cell

Component	Material	Elastic modulus (MPa)	Poisson's ratio
Battery case	Stainless steel	2.1 × 10^5^	0.3
Spring			
Spacer			
Sealing O-ring	PP	1500	0.42
Lithium disc	Lithium	4900	0.33
Separator	PP/PE/PP	344.4 (ref. 44)	0.4
Electrode	Silicon composite	1750 (ref. 45)	0.3
Copper foil	Copper	7 × 10^4^ (ref. 46)	0.3

**Fig. 13:**
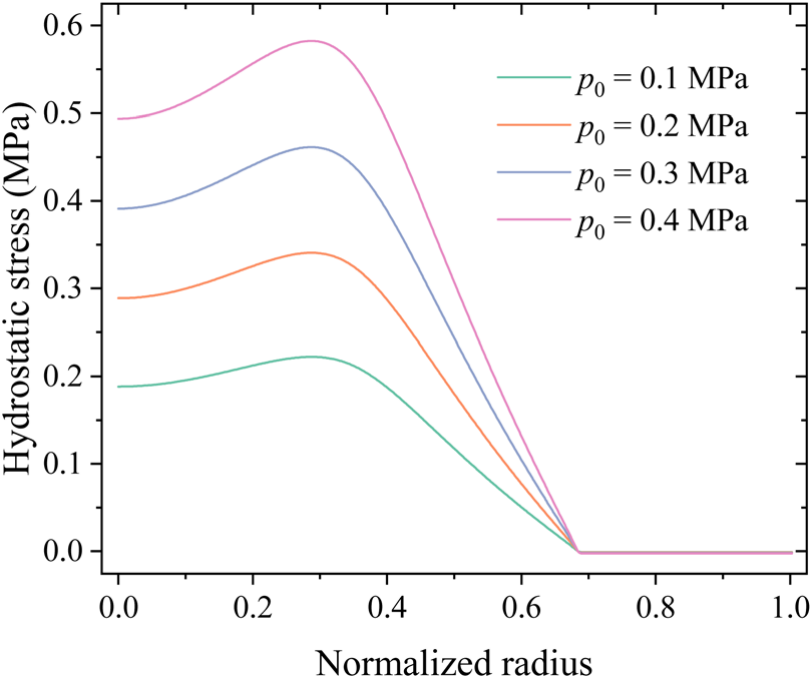
Distributions of hydrostatic stress in the middle surface of the electrode under different compressions.

Fig. 13 shows the distributions of hydrostatic stress in the middle surface of the electrode under different compressions. The numerical results indicate that external loads do introduce hydrostatic stress in most regions of the electrode, *i.e.*, mechanical load is indeed transmitted to the electrode.
